# Chemical composition, antioxidant, antimicrobial and Antiproliferative activities of essential oil of *Mentha spicata* L. (Lamiaceae) from Algerian Saharan atlas

**DOI:** 10.1186/s12906-018-2274-x

**Published:** 2018-07-03

**Authors:** Sanaa K. Bardaweel, Boulanouar Bakchiche, Husam A. ALSalamat, Maria Rezzoug, Abdelaziz Gherib, Guido Flamini

**Affiliations:** 1 Department of Pharmaceutical Sciences, School of Pharmacy, University of Jordan, Queen Rania Street, Amman, 11942 Jordan; 2 Laboratory of Process Engineering, Faculty of Technology, Laghouat University, 03000 Laghouat, Algeria; 3 Dipartimento di Farmacia, Università di Pisa, Via Bonanno 6, 56126 Pisa, Italy

**Keywords:** *M. spicata*, Essential oil, Chemical composition, Antioxidant, Antimicrobial, Antiproliferative

## Abstract

**Background:**

*Mentha spicata* (*M. spicata*) is a member of Lamiaceae that spreads mainly in the temperate and sub-temperate zones of the world. It is considered as a rich source of essential oils, which is widely used in pharmaceutical industries and food production. The objectives of the current study were to evaluate chemical composition, antioxidant, antimicrobial and antiproliferative activities associated with the essential oil of *M. spicata* cultivated in Algerian Saharan Atlas.

**Methods:**

The aerial parts of *M. spicata* were subjected to hydrodistillation to produce the oil. Chemical identification of the oil composition was conducted by GC and GC-MS analyses. The antioxidant activity of the hydrodistilled oil was studied using DPPH, ABTS radical scavenging and ferric-reducing power assay. Antimicrobial potential was characterized against two microorganisms, signifying Gram positive, and Gram negative bacteria, and one Candida species. The microdilution method was employed to determine the minimum inhibitory concentration (MIC). The oil’s antiproliferative effects against three human tumor cell lines were also investigated using the MTT assay, and the toxic doses that yielded 50% reduction of cell viability (LD_50_) were reported.

**Results:**

Chemical analysis of the essential oil composition revealed 44 unique compounds with oxygenated monoterpenes (67.2%), followed by monoterpene hydrocarbons (20.8%), as the most abundant chemical components. Essential oil of *M. spicata* demonstrated moderate antioxidant activities as well as moderate to weak antimicrobial activities with best susceptibility observed for Gram positive bacteria towards the oil. In addition, anticancer activities that are associated with the oil against three human cancer cell lines were observed with LD_50_ values of 324 μg/mL, 279 μg/mL, 975 μg/mL against T47D, HCT-116 and MCF-7 cell lines, respectively.

**Conclusion:**

The results suggest that *M. spicata* essential oil may have potential value as a bioactive oil, for nutraceutical and medical applications, with its antioxidant, antimicrobial and antiproliferative activities.

## Background

Recently, there has been renewed interest in the pharmacological features of aromatic plants that can be ascribed to their essential oils. Commonly, essential oils have been reported as bioactive secondary metabolites. Biological activities, comprising analgesic, antiseptic, sedative, spasmolytic, anesthetic and anti-inflammatory effects have been all reported for essential oils extracted from various plants [1, 2]. Although essential oils from aromatic and medicinal plants have been acknowledged for their diverse bioactivities since antiquity, it is only recently that methodical studies have been inaugurated to thoroughly investigate their bioactive adequacy in relation to their phytochemical characteristics [3, 4]. Traditionally, various plant parts have been employed to obtain essential oils, including flowers, leaves, seeds, roots, stems, bark and wood [5]. These oils have been widely utilized in medicines as active ingredients or in cosmetics as fragrances [6–9].

The genus Mentha (Lamiaceae family) includes more than 30 species of herbaceous perennial plants [10–13]. Mints are primarily habitants of temperate provinces of the world and exhibit significant discrepancy in their growth features, aromas and natural habitations [10, 13]. Several species of Mentha are used in folk medicine as flavoring agents and as herbal medicines for various therapeutic applications [14, 15]. Interestingly, Mentha essential oils were recognized as rich in oxygenated monoterpenes [14]. Controlled by the nature of their chief components, Mentha essential oils have been frequently used in different commercial and pharmaceutical products [16, 17].

*Mentha spicata* L. is distinguished by its characteristic essential oil of commercial and therapeutic importance. It is broadly cultivated in many regions worldwide to commercially produce its essential oil [18–20]. Lately, *M. spicata* has gained increased scientific interest considering other potential uses of its essential oil, predominantly, as antimicrobial and antioxidant bioactive natural extract [21].

To the best of our knowledge, this is the first study investigating the chemical composition and the biological activity profile of essential oil of *M. spicata* cultivated in the Algerian Saharan Atlas (Laghouat region).

## Methodology

### Collection and pretreatment of plant material

Aerial fragments of wild *M. spicata* L. (Lamiaceae) at full flowering phase were collected in May 2017, from the Laghouat region (Algeria). The samples were identified at the Department of Agronomy, Faculty of Science, University of Laghouat (Algeria), and the voucher specimens (LGP Ms1–3/05/17) were deposited at the laboratory of Process Engineering, University of Laghouat. The dried plant material was stored in the laboratory at room temperature (25 °C), protected from light, until extraction.

### Isolation of the essential oil

Essential oil was extracted from the dried aerial parts of *M. spicata* by hydro-distillation using an apparatus of Clevenger type. The extraction was carried out for 4 h to mix 200 g of plants in 1500 mL of distilled water. The extracts were dried with anhydrous sulphate and concentrated under reduced pressure by rotatory evaporator to evaporate water. The pure oil was stored at − 4 °C until further analyzed. The essential oils yield is demonstrated by the oil quality (in mL) obtained for 100 g of dried material.

### Physicochemical properties

The physicochemical properties, namely refractive index, specific gravity, color, solubility and acid number, were determined following Association of Official Analytical Chemists (AOAC) standard methods [22]. The refractive index and specific gravity were measured at 20 °C.

### Analysis of the essential oil

The essential oil chemical composition assessments and the identification of the main constituents were conducted by GC and GC-MS analyses. GC/MS analyses were performed with a Varian CP-3800 gas-chromatograph equipped with a DB-5 capillary column (30 m × 0.25 mm, coating thickness 0.25 μm) and a Varian Saturn 2000 ion trap mass detector. Analytical conditions were as follows: injector and transfer line temperatures 220 °C and 240 °C respectively; oven temperature programmed from 60 °C to 240 °C at 3 °C/min; carrier gas helium at 1 mL/min; injection of 0.2 μL (10% hexane solution); split ratio 1:30. The identification of the constituents was based on the comparison of the retention times with those of authentic samples, comparing their linear retention indices relative to the series of *n*-hydrocarbons, and on computer matching against commercial [23] and home-made library mass spectra built up from pure substances and components of known oils and MS literature data [23, 24].

### Antioxidant activity

#### DPPH radical scavenging activity

The antioxidant activity of the essential oil based on the scavenging activity of the stable 1,1-diphenyl-2- picrylhydrazyl free radical was determined by the method described by [25].A volume of 0.1 mL of essential oil from *M. spicata* prepared at different concentrations was mixed with 1.9 mL of 60 μM DPPH methanol solution. The disappearance of the DPPH was measured at 517 nm after 30 min of incubation at room temperature. The inhibition percentage of the DPPH radical by the essential oil was estimated using the following equation: Scavenging effect (%) = [100*(A_C_ -A_S_/Ac)], where Ac is the absorbance of the control reaction (containing all reagents except the test sample) and A_S_ the absorbance of the tested sample. The concentration of oil that could scavenge 50% of the DPPH radicals (IC_50_) was calculated. Ascorbic acid and BHT were used as standards for comparison.

#### ABTS radical scavenging activity

ABTS radical scavenging activity of *M. spicata* essential oil was measured by the ABTS cation decolorization assay as described by [26] with some modifications. The ABTS radical cation (ABTS^•+^) was produced by reaction of 7 mM stock solution of ABTS with 2.45 mM potassium persulfate and allowing the mixture to stand in dark at room temperature (25 °C) for 12 h before use. The ABTS^•+^ solution was diluted with methanol to give an absorbance of 0.7 ± 0.01 at 734 nm.20 μL of essential oil from *M. spicata* prepared at different concentrations were allowed to react with 1980 μL of the ABTS^•+^ solution and the absorbance was measured at 734 nm after 6 min. The scavenging rate and IC_50_ value were calculated using the equation described for DPPH assay. Ascorbic acid and BHT were used as standards for comparison.

#### Ferric-reducing power assay (FRAP assay)

The reducing power of the essential oil was measured by making use of the method described by [27] with some modifications. 0.1 mL of various concentrations of essential oil in methanol was taken separately and mixed with 2 mL of 0.2 M sodium phosphate buffer (pH 6.6). The diluted sample was then mixed with 2 mL of 1% potassium ferricyanide[K_3_Fe(CN)_6_] and the mixture was incubated at 50 °C for 20 min. 2 mL of 10% trichloroacetic acid (TCA) was added to the mixture and centrifuged at 3000 rpm for 10 min. 2 mL of the supernatant solution was mixed with 2.5 mL of distilled water and 0.5 mL of 1% ferric chloride (FeCl_3_), and the absorbance was measured at 700 nm. The IC_50_ value is the effective concentration at which the absorbance is 0.5 and is obtained by the equation described for DPPH assay. Ascorbic acid and BHT were used as standards for comparison.

### Antimicrobial activity

#### Microbial strains and growth conditions

*Staphylococcus epidermidis* ATCC 12228; a representative of Gram positive, *Escherichia coli* ATCC 29425; a representative of Gram negative and *Candida glabrata* ATCC 22553; fungi, were obtained from the Microbial Culture Collection Center of Medicine School at The University of Jordan, Jordan. Microorganisms were grown in nutrient broth medium and incubated, with shaking, at 37 °C and at 33 °C, for bacteria and candida respectively.

#### MIC determination

The minimum inhibitory concentration (MIC) determination, designated as the least concentration at which more than 80% of the microbial growth is inhibited, was carried out in 96 flat bottom microtiter plates (TPP, Switzerland) according to the microdilution method [28, 29]. Each microorganism was inoculated in each microtiter plate well at an inoculum size of 1 × 10^5^ CFU mL^− 1^. Positive controls, Ampicillin for bacteria and Amphotericin B for fungi, and a negative control of the vehicle (DMSO), were prepared under the same experimental conditions. Bacterial testing plates were incubated for 48 h at 37 °C, whereas Candida plates were incubated for 48 h at 33 °C, with shaking. To evaluate microbial growth, optical densities were measured at 600 nm (OD_600_) using a Microplate Reader (Palo Alto, CA, USA).

### Antiproliferative activity

#### Cells and cell culture conditions

The human colon cancer HCT-116, the human breast adenocarcinoma MCF-7, and the human ductal carcinoma T47D cell lines were purchased from the American Type Culture Collection (Rockville, MD, USA). Cells were grown and maintained in Dulbecco’s modified Eagle’s medium (DMEM, Gibco, Waltham, MD, USA) supplemented with 10% fetal bovine serum (FBS), and penicillin (100 U/mL) and were incubated at 37 °C in a humidified atmosphere of 95% O_2_ and 5% CO_2_. Cell cultures were passaged every 2–3-days or whenever reaching 80% confluent. Cells were seeded at a density of 6-8 × 10^3^ cells/well in 96-well plates and incubated for 24 h for adhesion.

#### Cell proliferation assay (MTT)

The MTT colorimetric assay was employed to evaluate cell proliferation as previously described [5]. In brief, test samples were prepared by dissolving the essential oil in DMSO followed by further dilution with DMEM medium to reach the desired final concentration. The final DMSO concentration in the assay was kept as low as 0.1%. Test samples containing the desired concentration of the essential oil were added to the wells. After exposure period of 48 h, MTT solution was applied into each well and incubated for 4 h at 37 °C. Afterward, DMSO was added to each well to solubilize the purple formazan crystals formed. Then, absorbance was read using a micro-plate reader at 570 nm. Doxorubicin was used as the positive control and 0.1% DMSO in DMEM media as solvent control.

### Statistical analysis

All experiments were conducted in triplicates and results are expressed as mean ± standard deviation (SD), being analysed using a Student’s t-test, with α = 0.05. The analyses were carried out using IBM SPSS Statistics for Windows, Version 22.0. (IBM Corp., Armonk, New York, USA).

## Results

### Chemical composition of the essential oil

The essential oil was extracted by the hydrodistillation of the dried aerial parts of *M. spicata* from the Laghouat region (Algeria), and was analyzed by GC-MS. The yield of the oil was 1.04 mL per 100 g plant material, soluble in 80% alcohol, with a pale-yellow color and persistent aromatic-spicy odor. The refractive index, specific gravity, and acid value of the essential oil were 1.48; 0.88 and 1.30, respectively, which indicate high quality and purity of the volatile oil.

As shown in Table 1, analysis of the essential oil resulted in identification of 44 compounds, representing 98.40% of the total oil, with carvone (49.5%), limonene (16.1%), 1,8-cineole (8.7%), *cis*-dihydrocarvone (3.9%), β-caryophyllene (2.7%), germacrene D (2.1%) and β-pinene (1.1%) were the predominating components. The most abundant chemical structure within components was the oxygenated monoterpenes (67.2%), followed by monoterpene hydrocarbons (20.8%), sesquiterpene hydrocarbons (7.5%) and oxygenated sesquiterpenes (1.2%).

**Table 1:** Chemical composition of the essential oil from aerial parts of *M. spicatai*

Constituents	LRI	%Area	Method of Identification ^a^
α-pinene	941	0.7	MS; LRI; RC
sabinene	977	0.6	MS; LRI; RC
β-pinene	982	1.1	MS; LRI; RC
myrcene	993	0.8	MS; LRI; RC
3-octanol	994	0.3	MS; LRI; RC
α-terpinene	1020	0.3	MS; LRI; RC
*p*-cymene	1028	0.2	MS; LRI; RC
**limonene**	1032	16.1	MS; LRI; RC
**1,8-cineole**	1034	8.7	MS; LRI; RC
*(Z)*-β-ocimene	1042	0.3	MS; LRI; RC
*(E)*-β-ocimene	1052	0.1	MS; LRI; RC
γ-terpinene	1063	0.4	MS; LRI; RC
*cis*-sabinene hydrate	1070	0.8	MS; LRI; RC
terpinolene	1090	0.2	MS; LRI; RC
linalool	1101	0.2	MS; LRI; RC
nonanal	1104	0.1	MS; LRI; RC
*cis-p*-menth-2-en-1-ol	1123	0.2	MS; LRI
δ-terpineol	1172	0.5	MS; LRI
4-terpineol	1179	1.5	MS; LRI; RC
α-terpineol	1191	0.3	MS; LRI; RC
*cis*-dihydrocarvone	1195	3.9	MS; LRI; RC
*trans*-carveol	1219	0.2	MS; LRI; RC
*(Z)*-3-hexenyl isovalerate	1238	0.9	MS; LRI; RC
pulegone	1239	0.5	MS; LRI; RC
**carvone**	1244	49.5	MS; LRI; RC
dihydroedulan IA	1292	0.1	MS; LRI
isodihydrocarvyl acetate	1329	0.6	MS; LRI
*cis*-carvyl acetate	1364	0.3	MS; LRI; RC
β-elemene	1392	0.3	MS; LRI
*(Z)*-jasmone	1395	0,3	MS; LRI; RC
β-caryophyllene	1419	2.7	MS; LRI; RC
β-copaene	1430	0.2	MS; LRI
aromadendrene	1440	0.1	MS; LRI; RC
α-humulene	1455	0.2	MS; LRI; RC
*cis*-muurola-4(14),5-diene	1463	0.3	MS; LRI
germacrene D	1482	2.1	MS; LRI
bicyclogermacrene	1496	0.7	MS; LRI
germacrene A	1505	0.7	MS; LRI
δ-cadinene	1524	0.2	MS; LRI
spathulenol	1577	0.2	MS; LRI
caryophyllene oxide	1582	0.3	MS; LRI; RC
1,10-di-*epi*-cubenol	1615	0.2	MS; LRI
T-cadinol	1641	0.3	MS; LRI
T-muurolol	1642	0.2	MS; LRI
Monoterpene hydrocarbons		20.8	
Oxygenated monoterpenes		67.2	
Sesquiterpene hydrocarbons		7.5	
Oxygenated sesquiterpenes		1.2	
Other Compounds		1.7	
Total identified		**98.4**	

^a^Method of identification: *MS* mass spectrum, *LRI* linear retention index, *RC* reference compound

### Antioxidant activity

The antioxidant activities of essential oil of *M. spicata* were determined by DPPH, ABTS and FRAP. The results are summarized in Table 2. In DPPH, the assessed sample was able to reduce the stable violet DPPH radical to the yellow DPPH-H, reaching 50% of reduction with IC_50_ value of (3450 ± 172.5 μg/mL). Relative to the potent antioxidant agents, ascorbic acid (IC_50_ = 2.22 ± 0.04 μg/mL) and BHT (15.20 ± 0.02 μg/mL), *M. spicata* exhibits weak antioxidant potential. Interestingly, essential oil of *M. spicata* grown in Iran demonstrated better antioxidant activity (81.66 μg/mL) [30], similar to that reported from Turkey (77.40 μg/mL) [31] and Egypt (63.80 μg/mL) [32].

**Table 2:** Antioxidant activities of aerial parts of *M. spicate* essential oil

	Scavenging activity IC_50_ (μg/mL)		
Parameters	DPPH	ABTS	FRAP
Essential oil	3450 ± 172.5^a^	40.2 ± 0.2^a^	215 ± 4.50^a^
Ascorbic acid	2.22 ± 0.04^b^	0.82 ± 0.01^b^	2.2 ± 0.1^b^
BHT	15.20 ± 0.02^b^	1.42 ± 0.05^b^	18.2 ± 0.5^b^

Each value in the table is represented as mean ± SD (*n* = 3). Means not sharing the same letter are significantly different at *P* < 0.05 probability level in each column

The total antioxidant capacity was also characterized by neutralization of the radical cation of *2,*2*′-*azino*-*bis(3*-*ethylbenzo thiazoline*-*6*-*sulphonic acid). The ABTS activity obtained for *M. spicata* essential oil (IC_50_ value of 40.2 ± 0.2 μg/mL) was more potent than those described [30] for *M. spicata* grown in Iran (IC_50_ value of 173.80 μg/mL)*.*

For Ferric reducing power, the presence of reducing agents in the extracts of plants causes thereduction of Fe^+ 3^- ferricyanide complex to the ferrous form. Therefore, Fe^+ 2^ can be assessed byfollowing the increase in the density of blue color in the reaction medium at700 nm [33]. *M. spicata* essential oil showed antioxidant activity with an IC_50_ value of 215 ± 4.50 μg/mL while ascorbic acid and BHT as a positive control showed IC_50_ values of 2.2 ± 0.1 and 18.2 ± 0.5 μg/mL, respectively. These results suggest that the essential oil of *M. spicata* has a remarkable potency to donate electron to reactive free radicals, converting them into more stable non-reactive species and terminating the free radical chain reaction.

### Antimicrobial activity

The essential oil of *M. spicata* was examined for its antimicrobial activity potential against a panel of pathogenic microorganisms including Gram positive, Gram negative, and fungi (Table 3). *Mentha spicata* oil demonstrated variable level of antimicrobial activity against all examined microorganisms. Results obtained from minimum inhibitory concentration (MIC) determination indicated that the essential oil was most active against the Gram-positive *Staphylococcus epidermidis* with MIC value of 32 μg\mL.

**Table 3:** Antimicrobial activity of aerial parts of *M. spicata* essential oil measured by MIC (μg/mL)

	Antimicrobial activity MIC (μg/mL)		
Microorganism	*Staphylococcus epidermidis*	*Escherichia coli*	*Candida glabrata*
Essential oil	32	64	256
Ampicillin	2	4	–
Amphotericin B	–	–	2

### Antiproliferative activity

**Fig. 1:**
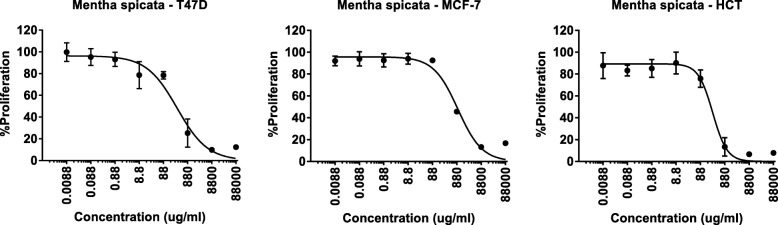
Antiproliferative activities of aerial parts of *M. spicate* essential oil on three cancer cell lines, exposure time 48 h

To explore other possible biological activity of the essential oil, *M. spicata* hydrodistilled oil was further evaluated for its in vitro antiproliferative characteristics on three human cancer cell lines, including human breast adenocarcinoma MCF-7 cell line, the human ductal breast epithelial tumor T47D cell line, and the human colon cancer HCT-116. Antiproliferative activities associated with *M. spicata* oil on the examined cell lines are presented in Fig. 1. The concentration of the oil at which survival was reduced by 50% (LD_50_) was calculated based on the dose-dependent curves and were 324 ± 81 μg/mL, 279 ± 52 μg/mL, and 975 ± 156 μg/mL for T47D, HCT-116 and MCF-7 cell lines, respectively. Positive control Doxorubicin LD_50_ values were in the range of 5–25 μg/mL. The anticancer activities that were observed on the three cancer cell lines under examination can be attributed to the significant abundance of some of the most naturally active anticancer components of the oil, such as1,8-cineole and limonene [34].

## Discussion

The data on the chemical composition of *Menta spicata* L. (Lamiaceae) essential oil cultivated in the Algerian Saharan Atlas has been reported for the first time. Previous studies have shown that monoterpenes, including carvone, limonene, and 1,8-cineole, to be the main components of the essential oil from *M. spicata* [35–37]. Similar chemical composition of *Mentha spicata* essential oil from Bejaia location in Algeria was reported [38]. Interestingly, the essential oil under investigation in this study appears to be richer with some key constituents such as 1,8-cineole and oxygenated monoterpenes. A review of the literature available on this topic reveals that many studies have already been published on *M. spicata* essential oils chemical composition [39], nevertheless there are no reports on the composition of *M. spicata* from Algerian Saharan Atlas region. Noteworthy, the composition of these volatile oils varies according to the countries, or the places in the same country. These differences seem to depend on climate changes and other factors like the method and the time of extraction, which can influence essential oil composition [40, 41].

In an effort to characterize the biological activity of *M. spicata* essential oil, the antimicrobial activity of the essential oil was examined in the present study. Interestingly, reports from literature on the association between the essential oil phytochemical composition and its antimicrobial activity are found to be in great consistency with the findings of the present study. In particular, oxygenated monoterpenes were evidently reported as potent antimicrobial agent in the composition of several essential oils [42]. In addition, 1,8-cineole and sesquiterpenes were shown to exhibit considerable antimicrobial activity against a wide range of Gram-positive and Gram-negative bacteria [42, 43]. However, as essential oils contain multiple components, their antimicrobial activities are rather due to additive, synergistic or antagonistic effects of the individual constituents.

Additionally, the results of the ABTS and FRAP assays revealed that *M. spicata* essential oil possessed reasonable antioxidant activity in the two assays. This could be attributed to the presence of high quantity of carvone in sample. In addition, it has been previously reported that compounds such as monoterpenes, and oxygenated monoterpenes may have antioxidant potential equivalent to that of a strong antioxidant [44]. Nonetheless, non-phenolic terpenoids were also reported to have substantial antioxidant potential [45]. Moreover, the observed antioxidant activity might be attributed to several other components of essential oils, including α-terpinene and 1,8-cineole [5, 46]. Accordingly, the abundance of potent antioxidants ingredients in the chemical composition of *M. spicata* oil would reasonably rationalize its moderate antioxidant activity.

Despite the endogenous antioxidant defense mechanisms, cell damage from oxygen free radicals is ubiquitous. Evidence has begun to accumulate supporting the critical role of free radicals in the initiation and development of cancer [47]. Literature reports have demonstrated that antioxidants may slow or possibly counteract cancer development and progress [48]. In this study, the observed anticancer activity of *M. spicata* oil might be linked to its ability to neutralize free radicals in cancer cells. Tumor cells survive by complex molecular signaling pathways. Targeting the different signaling pathways, by multiple agents, to block, retard, or reverse the tumorigenesis has been an effective strategy in cancer chemoprevention and chemotherapy. Essential oils are among of the most beneficial plant products utilized in medicine and complementary treatment approaches. Since *M. spicata* essential oil is generally recognized as safe, and thus widely used in food additives and cosmetics, the results of this study support the use of this oil as a potential source of new active anticancer agent. In order to assess the applied value of this potential therapeutic application, further investigation into the different mechanisms of action of the multiple components of the oil should be carried out.

## Conclusion

In summary, results of the current study revealed that the essential oil of *M. spicata* demonstrated moderate bioactivity elucidated by its antioxidant, antimicrobial and anticancer potential. These results suggest that the bioactive oil can be beneficially employed in pharmaceutical industries as well as in food production technologies.
